# Effect of Wearing Personal Protective Equipment (PPE) on CPR Quality in Times of the COVID-19 Pandemic—A Simulation, Randomised Crossover Trial

**DOI:** 10.3390/jcm10081728

**Published:** 2021-04-16

**Authors:** Simon Rauch, Michiel Jan van Veelen, Rosmarie Oberhammer, Tomas Dal Cappello, Giulia Roveri, Elisabeth Gruber, Giacomo Strapazzon

**Affiliations:** 1 Institute of Mountain Emergency Medicine, Eurac Research, Viale Druso 1, 39100 Bolzano, Italy; Michiel.vanVeelen@eurac.edu (M.J.v.V.); Tomas.DalCappello@eurac.edu (T.D.C.); giuliaroveri.md@gmail.com (G.R.); giacomo.strapazzon@eurac.edu (G.S.); 2 Department of Anaesthesia and Intensive Care, “F. Tappeiner” Hospital, Via Rossini 5, 39012 Merano, Italy; 3 Southtyrolean Helicopter Emergency Medical Service, Via Lorenz Böhler 3, 39100 Bolzano, Italy; rosmarie.oberhammer@sabes.it (R.O.); elisabeth.gruber@sabes.it (E.G.); 4 Department of Anaesthesia and Intensive Care, Brunico General Hospital, Via Ospedale 11, 39031 Brunico, Italy

**Keywords:** cardiopulmonary resuscitation, chest compression, personal protective equipment, COVID-19, airborne disease transmission prevention, aerosol generating procedure

## Abstract

Cardiopulmonary resuscitation (CPR) is considered an aerosol-generating procedure. Consequently, COVID-19 resuscitation guidelines recommend the use of personal protective equipment (PPE) during resuscitation. In this simulation of randomised crossover trials, we investigated the influence of PPE on the quality of chest compressions (CCs). Thirty-four emergency medical service BLS-providers performed two 20 min CPR sequences (five 2 min cycles alternated by 2 min of rest) on manikins, once with and once without PPE, in a randomised order. The PPE was composed of a filtering facepiece 3 FFP3 mask, safety glasses, gloves and a long-sleeved gown. The primary outcome was defined as the difference between compression depth with and without PPE; secondary outcomes were defined as differences in CC rate, release and the number of effective CCs. The participants graded fatigue and performance, while generalised estimating equations (GEE) were used to analyse data. There was no significant difference in CC quality between sequences without and with PPE regarding depth (mean depth 54 ± 5 vs. 54 ± 6 mm respectively), rate (mean rate 119 ± 9 and 118 ± 6 compressions per minute), release (mean release 2 ± 2 vs. 2 ± 2 mm) and the number of effective CCs (43 ± 18 vs. 45 ± 17). The participants appraised higher fatigue when equipped with PPE in comparison to when equipped without PPE (*p* < 0.001), and lower performance was appraised when equipped with PPE in comparison to when equipped without PPE (*p* = 0.031). There is no negative effect of wearing PPE on the quality of CCs during CPR in comparison to not wearing PPE.

## 1. Introduction

The Coronavirus disease 2019 (COVID-19) pandemic is causing significant disruptions in healthcare systems worldwide, with about a hundred million confirmed cases and nearly 2.4 million deaths recorded as of mid-February 2020 [1]. Sultanian et al. have recently reported that 20.9% of patients in out of hospital cardiac arrest (OHCA) cases in Sweden have severe acute respiratory syndrome coronavirus type 2 (SARS-CoV-2) infections [2]. It is impossible for prehospital healthcare providers at the scene of an OHCA to rule out a SARS-CoV2-infection without delay. Cardiopulmonary resuscitation (CPR) is considered an aerosol-generating procedure [3], and unprotected exposure to droplets and airborne particles from an infected patient constitutes an infection risk. Despite a lack of direct evidence, the International Liaison Committee on Resuscitation (ILCOR) recommends that healthcare providers should therefore use personal protective equipment (PPE) for aerosol-generating procedures during resuscitation [4]. Currently, the European Resuscitation Council (ERC) recommends PPE for airborne precaution to consist of gloves, a long-sleeved gown, a filtering facepiece 3 (FFP3) or N99 mask/respirator (FFP2 or N95 if FFP3 is not available), and eye and face protection (full-face shield/visor, polycarbonate safety glasses or equivalent equipment) [5].

The act of performing adequate CCs is a strenuous activity, and one’s exercise capacity could be a limiting factor [6]. The effect of wearing face masks on physical exercise capacity due to increased breathing resistance is currently under debate [7]. There are little data published on the effect of PPE on the effectiveness or quality of CPR.

In this trial, we investigate the effect of wearing PPE (including an FFP3 face mask) on the quality of CCs during CPR performed by prehospital healthcare providers. We hypothesize that the quality of CCs delivered is not affected by wearing PPE.

## 2. Materials and Methods

The study was designed as a simulation randomised crossover trial and ran from 29 September to 14 October 2020 in Bolzano, Merano and Brunico (South Tyrol, Italy). The Ethics Committee review board of Bolzano, Italy, approved the study (protocol number 100–2020), and the study is registered on ClinicalTrials.gov (Protocol Record NCT04548934). The study was conducted in accordance with the principles of the Declaration of Helsinki.

### 2.1. Study Participants

Study participants were providers recruited from the prehospital emergency medical service organisation ‘Croce Bianca’ (South Tyrol, Italy). The inclusion criteria for study participants are as follows: participants must be aged between 18 and 65 years old, must have a valid certificate in BLS, must have no COVID-19 symptoms in the last four weeks before the test, must not be tested positive for SARS-CoV2, must have no quarantine or unprotected contact with COVID-19 patients in the last four weeks, must have a body temperature less than 37.5 °C on the test day and must have obtained written informed consent with respect to the study.

### 2.2. Study Protocol

Before the test, each participant practiced CCs on a manikin and was given visual and verbal feedback on the quality of their CCs. The participants were blinded neither to the intervention nor to the study purpose.

Each study participant performed two CPR sequences on a manikin, once with PPE and once without PPE in a randomised order. We used block randomization in order to guarantee that half of the participants started the sequences with PPE and that the other half of the participants started without PPE. Each sequence lasted 20 min and was composed of five cycles consisting of two minutes of CCs alternated by two minutes of no CCs (break), simulating a changeover, as is recommended by the current ERC guidelines [8]. Between the two CPR sequences (i.e., with and without PPE) a break of one hour for recovery was given. The PPE was composed of an FFP3 mask (Valmy Cyrano^®^ FFP3, Mably, France), safety glasses, gloves and a long-sleeved gown.

During both CPR sequences, the quality of CCs was measured using the manikin (Laerdal Resusci Anne QCPR, Stavanger, Norway), which was fitted with a standard compression spring that was connected to a tablet PC (Laerdal Simpad PLUS, Stavanger, Norway). The indicators of CC quality were comprised of compression depth, rate, release and the number of effective CCs, where compressions with a depth of greater than 50 mm at the correct position (mid-chest) were counted as effective.

Directly after each sequence, participants were asked by trained members of the study team to subjectively grade fatigue and performance using an eleven point (from 0 to 10) numeric rating scale (NRS), where 0 corresponded to minimal fatigue or performance and 10 corresponded to maximal fatigue or performance.

### 2.3. Study Outcomes

The difference between compression depths with and without PPE was defined as the primary outcome. Secondary outcomes were defined as differences in CC rate, release and the number of effective CCs. Additional outcome measures included the influence of gender, body weight, PPE sequence order and cycle number on CC quality. The subjective fatigue and participant performance evaluations, with and without PPE, were defined as tertiary study outcomes.

### 2.4. Statistical Analysis

According to the crossover design, which had an expected mean difference of 5 mm and a standard deviation of the difference of 9 mm (effect size 0.55), a sample size of 28 matched pairs was calculated as sufficient to evaluate the primary outcome at a significance level of 0.05 (two-sided) with 80% power [9].

Each 2 min cycle of CCs was divided into four 30 s timepoints, and the average of CC depth, rate, and release and the number of effective CCs per timepoint were considered dependent variables. To take into account the repeated measures of each participant (four 30 s timepoints for each of the five cycles), generalised estimating equations (GEE) were performed for each dependent variable to analyse whether the following factors had an effect: PPE (with or without), cycle number, timepoint, PPE sequence order (first or second), weight (two groups considering the median of 80 kg as the cut-off), gender, the interactions of timepoint and cycle number with PPE, gender and weight, and the interaction of PPE with gender.

The Wilcoxon signed rank test was performed to compare the participant’s subjective fatigue and performance when using or not using PPE, and Pearson correlation was implemented to correlate subjective fatigue and performance with the compression parameters. The Holm–Bonferroni method was used to correct *p*-values for multiple comparisons. SPSS version 26 (IBM Corp., Armonk, NY, USA) was used for statistical analysis, and *p* < 0.05 (two-sided) was considered statistically significant. The values are reported as mean ± standard deviation, except for estimates of the GEE as the mean (95% confidence interval).

## 3. Results

**Figure 1:**
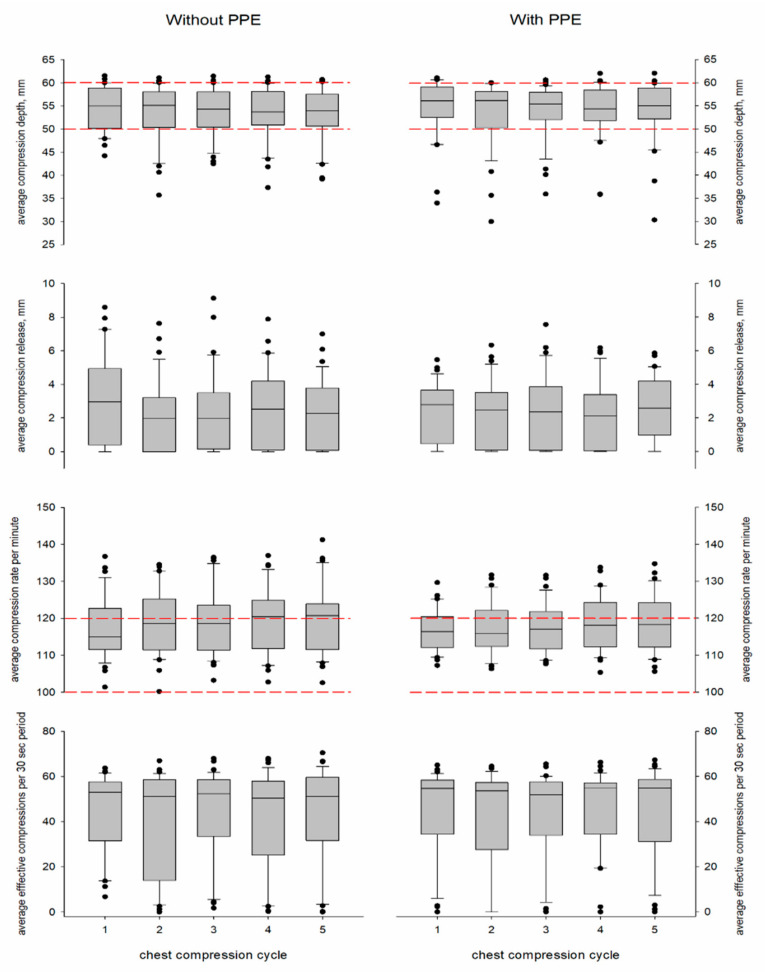
Boxplots of the evolution of chest compression depth, release, rate, and the number of effective chest compressions per consecutive 2 min cycle with and without personal protective equipment (PPE). The horizontal red dashed lines delineate the range of compression depth and rate recommended by the European Resuscitation Council guidelines [8]. The ● represents the outlier.

**Figure 2:**
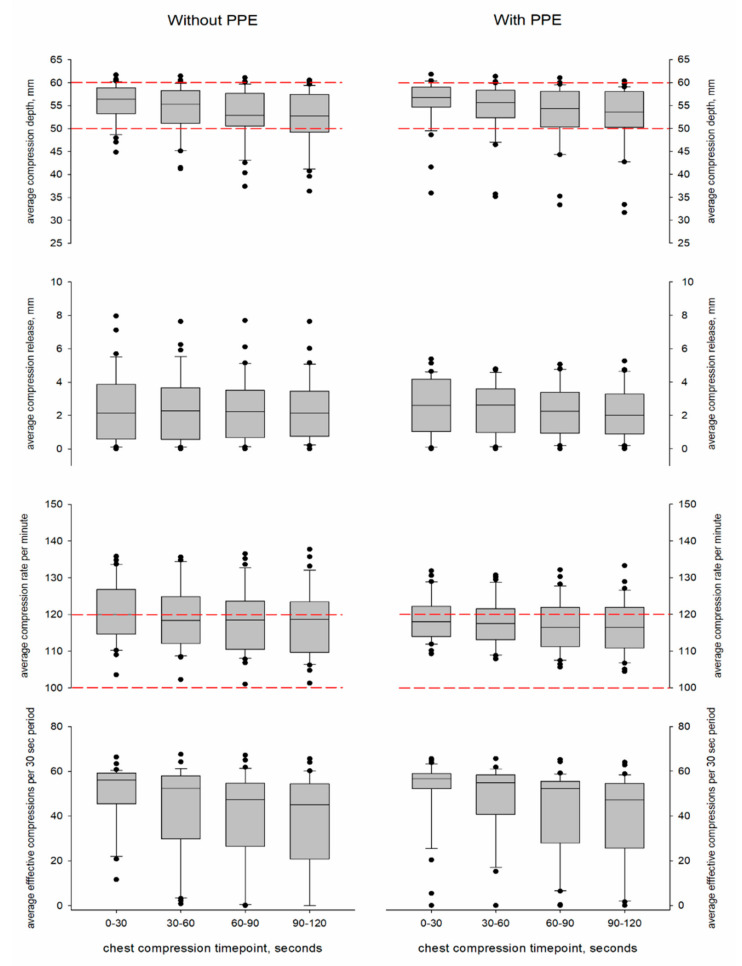
Boxplots of the evolution of chest compression depth, release, rate, and number of effective chest compressions per 30 s timepoint with and without personal protective equipment (PPE). The horizontal red dashed lines delineate the ranges of compression depth and rate that are recommended by the European Resuscitation Council guidelines [8].

Thirty-four participants were consecutively recruited, and all of them met the inclusion criteria. Participants’ baseline characteristics are shown in Table 1. All of the participants completed two 20 min sequences of CCs with and without PPE in a randomised sequence order. Due to technical problems, CC quality could not be recorded in three sequences (this consisted of two sequences with PPE and one sequence without PPE; the data are considered missing completely at random and discarded from the analysis); therefore, a total of 65 sessions were included in the analysis. The mean compression depth, release, rate, and number of effective CCs with and without PPE is shown in Table 2. Evolutions of the CC quality parameters per 2 min cycle and per 30 s timepoint during a cycle are illustrated in Figure 1 and Figure 2.

We found no effect of wearing PPE with respect to compression depth, release, rate and number of effective CCs (Table 3). Independent of PPE, the estimated means showed deeper compression during the first cycle (54 (52–56) mm) in comparison to the second (53 (51–55) mm, *p* = 0.001) and fifth cycles (53 (51–55) mm, *p* = 0.011), and an increasing rate from the first to the fifth cycles (117 (115–119) vs. 119 (117–122) compressions per minute, *p* < 0.001). In the 30 s timepoint analysis, the estimated means of compression depth, rate and number of effective compressions showed a decreasing trend that was not dependent on PPE: comparing the first 30 s with the last 30 s, depth decreased from 55 (54–57) mm to 52 (50–54) mm (*p* < 0.001), the rate decreased from 120 (117–122) to 117 (115–120) compressions per minute (*p* < 0.001) and the number of effective CCs decreased from 50 (45–56) to 37 (31–44) (*p* < 0.001). The effect of wearing PPE on compression parameters was not different between females and males (Table 3).

The participants appraised higher fatigue with PPE in comparison to without PPE (6.7 ± 1.8 vs. 5.0 ± 2.1 NRS, *p* < 0.001) and lower performance with PPE in comparison to without PPE (6.8 ± 1.4 vs. 7.3 ± 1.1 NRS, *p* = 0.031). Subjective fatigue and performance were not correlated with compression parameters (Table 4).

## 4. Discussion

We found no effect of wearing PPE (including an FFP3 mask) on CC quality during manikin CPR. We found a statistically significant difference in CC depth and rate over time, both during the entire 20 min sequence and during the single two-minute cycle. These differences were, however, similar for both sequences (with and without PPE) and presumably of no clinical significance, as virtually all delivered compressions were within the limits of resuscitation guidelines. Even though the participants experienced increased subjective fatigue and decreased subjective performance while wearing PPE, this did not reflect on the objective physical performance during the delivery of CCs. The perceived exertion of performance and reduced performance could be due to increased airflow resistance while breathing through an FFP2 or FFP3 mask [10].

Most studies that have investigated the effect of mask-wearing during exercise on physiological changes and subjective symptoms found only a minor physiological burden but relevant subjective disturbances [11,12,13]. This is consistent with our findings. Moreover, earlier studies assessing subjective performance during CCs confirm the inability of participants to self-estimate the adequate quality of CCs [14,15].

Our findings contradict a systematic review that included five trials and that subsequently concluded that the use of PPE compromises the quality of CCs during CPR [16]. However, this review has several limitations and its applicability to the current COVID-19 pandemic seems to be questionable. For instance, only one out of the five included studies was performed during the COVID-19 pandemic and, more importantly, in four out of the five trials, cumbersome Level B or level C HAZMAT full-face air purification devices were used. Moreover, only one trial had a clinically relevant difference regarding CC rate and depth between the PPE and no-PPE arm [16]. However, in this trial depth was inadequate both in the control (no-PPE) arm as well as in the intervention (PPE) arm (49.3 ± 6.9 mm versus 42.5 ± 6.8 mm, respectively) and the statistically significant difference in the mean CC rate was of dubious clinical relevance (105.4 ± 8.3 versus 98.1 ± 8.9 compressions per minute in the control arm and in the PPE arm, respectively) [17]. In a recent manikin study by Malysz et al., an insufficient compression depth (median 40 mm) was found during a 2 min cycle of CCs on a manikin while wearing PPE for aerosol-generating procedures [18]. Based on their findings, the authors argued that the CPR algorithm should be changed by reducing the duration of the CPR cycle for one rescuer from the current 2 min cycles to 1 min cycles when performing manual CCs. However, the trial did not have a control group (without PPE), and this renders the authors’ conclusions questionable.

Kienbacher et al. in their randomised controlled non-inferiority triple-crossover manikin study did not report any difference regarding the quality of CCs while wearing or not wearing PPE with an FFP2 mask, which is similar to our findings [19]. Moreover, the providers in their trial reported a subjectively higher physical strain, but physical exhaustion did not correlate well with the quality of CPR.

To our knowledge, our study is the first study comparing the quality of CCs while wearing an FFP3 mask. Our findings confirm that the use of PPE as recommended by the current COVID-19 resuscitation guidelines [5], even when wearing an FFP3 mask, does not alter CC quality during simulated manikin CPR compared to when not wearing PPE.

Our study has some limitations. In the first place, it is a manikin study with all its associated limitations. In the second place, we did not take into account the time needed to equip the PPE. Donning PPE can cause a significant delay in CPR initiation, which has associated negative patient outcomes. Finally, vision and communication between healthcare providers during CPR can be impaired by PPE, which could harm resuscitation outcomes [4,20]. Thus, high-fidelity simulation training with PPE should be performed regularly to optimise resuscitation outcomes.

## 5. Conclusions

There is no negative effect in wearing PPE for airborne disease transmission precaution on the quality of CCs delivered during CPR compared to when not wearing PPE. The results could help support evidence-based guidelines for resuscitation with respect to the particular circumstances of potential airborne disease transmission. However, wearing PPE during CPR leads to increased subjective fatigue and decreased subjective performance.

## Figures and Tables

**Table 1:** Baseline characteristics of the study participants.

Characteristic	Mean ± SD or *n* (%)
Age, years	40 ± 1.6
Female participants	13 (38%)
Height, m	1.75 ± 0.09
Body weight, kg	77 ± 14
BMI	24.3 ± 5.6
EMS experience, years	12.0 ± 10.7

SD—standard deviation; BMI—body mass index; EMS—emergency medical services.

**Table 2:** Compression depth, release, rate, and number of effective chest compressions with and without PPE. Values are given as mean ± standard deviation.

Parameter	Without PPE	With PPE
Compression depth, mm	54 ± 5	54 ± 6
Release, mm	2 ± 2	2 ± 2
Rate per minute	119 ± 9	118 ± 6
Effective chest compressions, number	43 ± 18	45 ± 17

PPE—personal protective equipment.

**Table 3:** Here, *p*-values of the factors are inserted in the generalised estimating equations# (GEE) for analysis of the compression parameters; the *p*-values are corrected by means of the Holm–Bonferroni method.

Compression Parameter	Intercept	PPE	Cycle Number	Timepoint	PPE Sequence Order	Weight	Gender	Timepoint * PPE	Timepoint * Gender	Timepoint * Weight	Cycle Number * PPE	Cycle Number * Gender	Cycle Number * Weight	PPE * Gender
Depth	**<0.001**	1.000	**0.002**	**<0.001**	1.000	0.604	1.000	1.000	1.000	1.000	1.000	0.054	1.000	1.000
Release	**<0.001**	1.000	0.462	0.054	1.000	1.000	1.000	0.826	1.000	0.695	0.989	†	†	1.000
Rate	**<0.001**	0.229	**<0.001**	**<0.001**	0.197	0.101	1.000	1.000	1.000	1.000	1.000	0.769	0.597	1.000
Effective CCs	**<0.001**	1.000	1.000	**<0.001**	1.000	1.000	1.000	1.000	1.000	0.541	1.000	1.000	0.540	1.000

PPE—personal protective equipment; CCs—chest compressions. * An asterisk between two factors denotes an interaction. # The specified distribution and link function were normal and identified for the chest compression depth and rate, Gamma and the logarithm for chest compression release, and Poisson and the logarithm for the number of effective chest compressions; the working correlation matrix was chosen by means of the Quasi-Likelihood under Independence Model Criterion (QIC) among 1-dependent, independent, unstructured and first-order autoregression (AR(1)) and was independent for chest compression depth, rate and release and AR(1) for the number of effective chest compressions. † Not calculated because of computational issues.

**Table 4:** The correlation between subjective fatigue and performance of chest compressions and compression parameters. Subjective fatigue and performance are evaluated using an eleven point numeric rating scale (0–10). The *p*-values are corrected by means of the Holm–Bonferroni method.

Subjective Parameter	PPE	Compression Parameter *	Correlation	*p*-Value
Fatigue	No	Depth	−0.121	1.000
		Release	−0.003	1.000
		Rate	0.213	1.000
		Effective CCs	−0.007	1.000
	Yes	Depth	−0.040	1.000
		Release	−0.041	1.000
		Rate	−0.016	1.000
		Effective CCs	−0.049	1.000
Performance	No	Depth	0.222	1.000
		Release	−0.274	0.977
		Rate	0.078	1.000
		Effective CCs	0.200	1.000
	Yes	Depth	0.080	1.000
		Release	0.103	1.000
		Rate	0.463	0.061
		Effective CCs	0.065	1.000

PPE—personal protective equipment; CCs—chest compressions. * Mean value of five cycles and four timepoints.

## Data Availability

The data presented in this study are available upon request from the corresponding author.
